# Shared genomic segment analysis in a large high-risk chronic lymphocytic leukemia pedigree implicates *CXCR4* in inherited risk

**DOI:** 10.20517/jtgg.2021.05

**Published:** 2021-06-15

**Authors:** Julie E. Feusier, Michael J. Madsen, Brian J. Avery, Justin A. Williams, Deborah M. Stephens, Boyu Hu, Afaf E. G. Osman, Martha J. Glenn, Nicola J. Camp

**Affiliations:** 1 Huntsman Cancer Institute, University of Utah, Salt Lake City, UT 84112, USA.; 2 Division of Hematology and Hematological Malignancies, Department of Internal Medicine, University of Utah, Salt Lake City, UT 84112, USA.

**Keywords:** Gene-mapping, chronic lymphocytic leukemia (CLL), linkage, *CXCR4*, shared genomic segment (SGS), pedigree, Utah Population Database (UPDB)

## Abstract

**Aim:**

Chronic lymphocytic leukemia (CLL) has been shown to cluster in families. First-degree relatives of individuals with CLL have an ~8 fold increased risk of developing the malignancy. Strong heritability suggests pedigree studies will have good power to localize pathogenic genes. However, CLL is relatively rare and heterogeneous, complicating ascertainment and analyses. Our goal was to identify CLL risk loci using unique resources available in Utah and methods to address intra-familial heterogeneity.

**Methods:**

We identified a six-generation high-risk CLL pedigree using the Utah Population Database. This pedigree contains 24 CLL cases connected by a common ancestor. We ascertained and genotyped eight CLL cases using a high-density SNP array, and then performed shared genomic segment (SGS) analysis - a method designed for extended high-risk pedigrees that accounts for heterogeneity.

**Results:**

We identified a genome-wide significant region (*P* = 1.9 × 10^−7^, LOD-equivalent 5.6) at 2q22.1. The 0.9 Mb region was inherited through 26 meioses and shared by seven of the eight genotyped cases. It sits within a ~6.25 Mb locus identified in a previous linkage study of 206 small CLL families. Our narrow region intersects two genes, including *CXCR4* which is highly expressed in CLL cells and implicated in maintenance and progression.

**Conclusion:**

SGS analysis of an extended high-risk CLL pedigree identified the most significant evidence to-date for a 0.9 Mb CLL disease locus at 2q22.1, harboring *CXCR4.* This discovery contributes to a growing literature implicating *CXCR4* in inherited risk to CLL. Investigation of the segregating haplotype in the pedigree will be valuable for elucidating risk variant(s).

## INTRODUCTION

Chronic lymphocytic leukemia (CLL) is the most common adult leukemia diagnosed in individuals of European ancestry in the United States (5.0/100,000)^[1]^. CLL has a strong heritable component, and first-degree relatives have a 7.5–8.5 fold elevated risk of developing CLL^[2–4]^. Therefore, a family-based design is relevant to consider for CLL. However, because CLL is relatively rare, this design presents a challenge in ascertainment of multi-case families. Translational relevance for successful family discoveries includes genetic counselling for at-risk family members and new avenues for understanding biological mechanism towards improved prevention and treatment.

An established family-based statistical approach is linkage analysis. Recombination events are estimated in families which localize regions, and risk haplotypes, which are inherited to affect family members. These haplotypes can subsequently be interrogated to identify specific variants involved in disease pathogenesis. This design boosts power for rarer risk alleles which are enriched in the family setting. In the study of familial CLL, five genome-wide linkage studies have been performed thus far^[5–9]^. Of these, only one locus has been proposed with genome-wide significance. Linkage analyses in 206 CLL families identified a significant peak at chromosome 2q21.2 (LOD-equivalent 3.11, *P* = 7.7 × 10^−5^), and a 1-LOD support interval defining a ~6.25 Mb locus at 2q21.2–2q22.1^[7]^. The locus contains the gene *CXCR4* (C-X-C chemokine receptor type 4) (at 2q22.1), of particular interest due to its key role in B cell lymphopoiesis and maintenance of immature B cells in the bone marrow.

Two family-based whole exome sequencing (WES) studies have been performed^[10,11]^. Rigorous statistical thresholds that account for the multiple phases and family-based expectations have not yet been defined for direct-to-sequencing family studies, hence these studies remain largely observational in nature. The prior studies focused on finding recurrent rare alleles in families, but the segregation of those alleles was not formally assessed (i.e., how probabilities for the findings are influenced by allele frequency, non-sharing in cases, and unaffected carriers). In a study of 59 small families, Goldin *et al.*^[10]^, focused on identifying coding variants recurrent within and shared across at least two families. They identified 6 families recurrent for an allele in *ITGB2* [rs2230531 at 21q22.3, minor allele frequency (MAF) = 0.007]. However, recurrence of this variant was not replicated in a follow-up study of *ITGB2* in 47 small families^[12]^. In a WES study of 66 families (no restriction made to sharing across multiple families), *ITGB2* variants were not identified, but four different coding variants were found to be recurrent within 7 different small families, all in genes involving the shelterin complex (four in *POT1*, one in *ACD* and two *TERF2IP* at 7q31.33, 16q22.1 and 16q23.1, respectively)^[11]^.

One family study used genome-wide genotype data to identify germline copy number variants (CNV) in CLL families occurring at regions known to be commonly aberrant in malignant CLL cells. This identified two germline CNVs: a mutation at 13q involving *DLEU7* and a gain at 6p including *IRF4*. Each was shared by a single CLL sib-pair^[13]^. These findings have yet to be replicated.

The scarcity of CLL family resources, heterogeneity across families and the likely complexity of the disease mechanism (multiple genes, multiple alleles, incomplete penetrance, and sporadic cases) leads to challenges in uncovering inheritable genetic abnormalities. Our goal was to identify CLL risk loci using unique resources available in Utah through the Utah Population Database (UPDB) to identify large, extended, high-risk pedigrees and a powerful new method specifically designed for large pedigrees and to address heterogeneity.

The UPDB includes a 16-generation genealogy of approximately 5 million people with at least one event in Utah that is record-linked to statewide cancer records since 1966 from the NCI Surveillance, Epidemiology, and End Results (SEER) Program Utah Cancer Registry (UCR) and state vital records^[14]^. Within the UPDB, ancestors whose descendants have an increased incidence of malignances as compared to internal cancer rate controls and years at risk can be identified and studied as high-risk pedigrees.

Shared genomic segment (SGS) analysis is a recombinant-based family analysis (“linkage-like”), developed to identify regions that segregate to cases in an extended high-risk pedigree^[15]^. When available to study, a single large pedigree can increase homogeneity, garner equivalent power to many small pedigrees, and be sufficient alone to declare genome-wide significance. However, full likelihood-based linkage approaches are intractable in very large pedigrees. Furthermore, traditional linkage methods are not robust to substantial intra-familial heterogeneity (sporadic cases), which must be accounted for in very large pedigrees. To combat this, SGS identifies long stretches of consecutive identity-by-state (IBS) alleles to infer shared inherited identity-by-descent (IBD) haplotypes. The algorithm iterates over (and corrects for) assessment of subsets of cases to account for possible sporadic cases. Overall, SGS is the ideal method for investigating disease risk loci shared by a common founder in large pedigrees.

Here, we use the UPDB to identify a six-generation high-risk CLL pedigree, the largest CLL family studied to-date. We performed SGS to identify inherited risk loci likely to harbor disease genes for CLL.

## METHODS

### Identification and ascertainment of the high-risk pedigree

**Figure 1.**
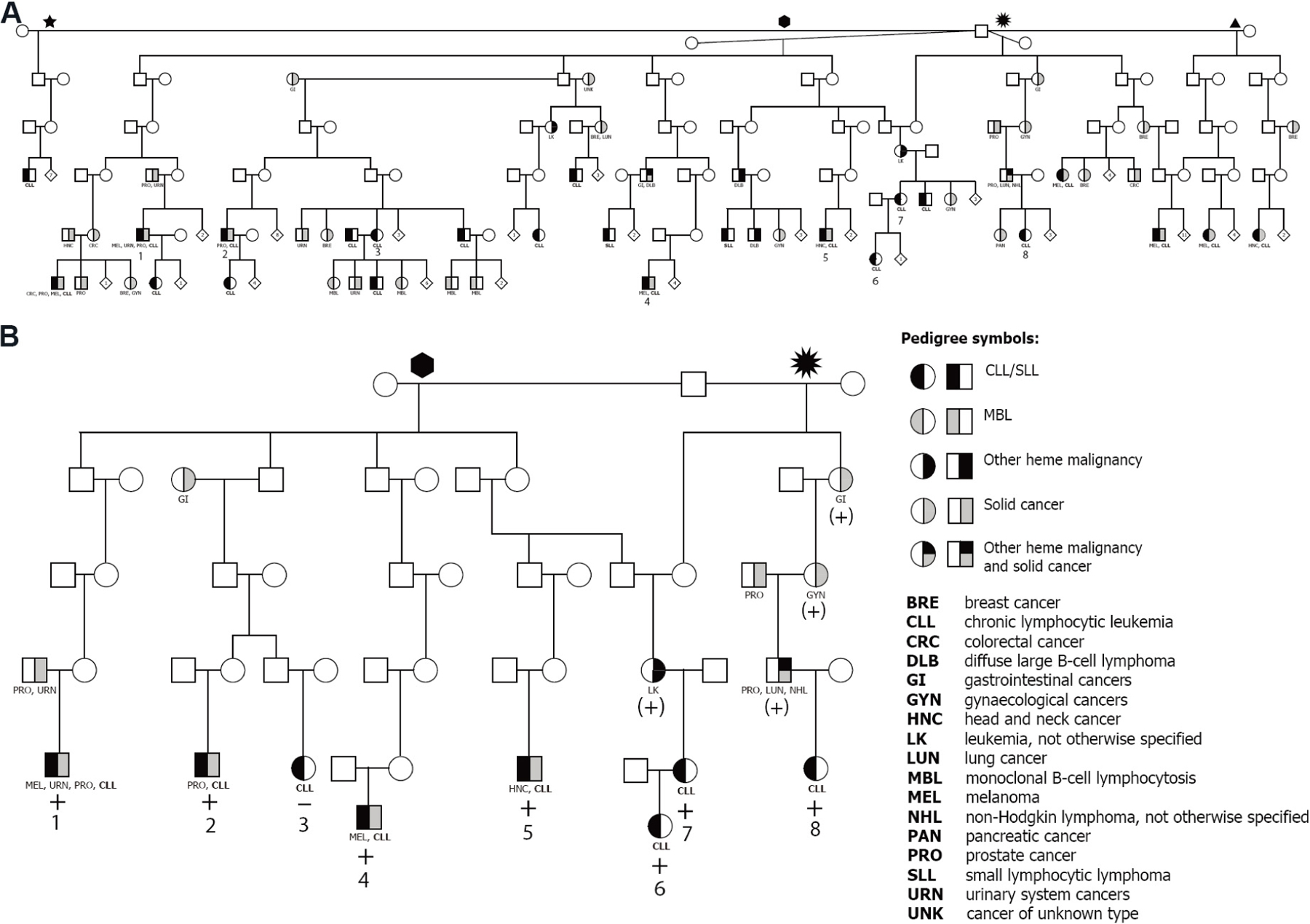
Extended high-risk chronic lymphocytic leukemia (CLL) pedigree from the Utah Population Database. (A) Full extent of CLL cases captured from the common ancestor, with four wives. (B) Reduced pedigree, terminating at each sampled CLL case and only containing the intermediate connecting relatives. “+” indicates sharing of the significant shared 2q22.1 region; “(+)” indicates obligate sharing with a heme malignancy or solid cancer; and “-” indicates non-sharing.

The UPDB was used to identify ancestors whose descendants showed a statistical excess of CLL (*P* < 0.05). Expectation was based on internal disease rates based on birth cohort, sex, birth place (in/outside Utah) and years at risk. These were considered high-risk CLL pedigrees. Once identified, living CLL cases within high-risk pedigrees were made aware of the study by representatives of the UCR, and those interested were invited to participate. Cases and family members wishing to be part of the study were subsequently enrolled by the study team, including informed consent, questionnaires and biospecimens. Individuals in 23 high-risk CLL pedigrees were enrolled as previously described^[16]^. Only one six-generation pedigree with 24 CLL cases contained sufficient meioses (m ≥ 15) between sampled CLL cases for SGS analysis^[17]^. Figure 1A illustrates all 24 cases in the pedigree. Figure 1B shows the reduced structure containing only the eight sampled CLL cases analyzed in the SGS analysis.

### Acquisition of materials and genotyping

Peripheral blood samples were processed to DNA. Individuals in the pedigrees were genotyped using the Illumina Human 610Q high-density single nucleotide polymorphism (SNP) array. Genotypes were called using standard Illumina protocols. Alleles were re-oriented to align with 1000 Genomes Project sequence data^[18]^. SNP quality control was performed alongside other project data using PLINK and included: SNP call-rate (95%), sample call-rate (90%), removal of monomorphic SNPs, and failure of Hardy-Weinberg equilibrium (*P* < 1.0 × 10^−5^)^[19–22]^. After quality control, 555,091 autosomal SNPs were available for SGS. The average age at diagnosis for these eight sampled cases was 61.5 (min 46, max 72). The average overall survival time for the five cases who subsequently died was 11.2 years (min 4.8, max 15.9).

### Shared genomic segment analysis

A detailed explanation of the SGS method, including optimization over subsets and determination of the genome-wide significance threshold has been described elsewhere^[15]^. Briefly, a segment is defined as the stretch of consecutive SNPs shared IBS by a subset of cases. Sharing is assessed for each subset (*n* ≥ 2) of cases in the pedigree. A segment is broken when two cases in a subset are opposite homozygotes and thus cannot share. At each position across the genome, the optimal segment across subsets is determined (smallest *P*-value). Together these become the genome-wide optimal results.

Nominal significance for each segment is established empirically. Simulated genotype configurations under the null hypothesis of no linkage are generated using a gene-drop procedure. This involves random assignment of chromosomes to pedigree founders (individuals in the pedigree without parents) based on a linkage disequilibrium map from the 1000G using graphical modeling^[23]^. The principles of Mendelian inheritance are then used to “drop” the chromosomes through the pedigree structure with recombination occurring according to the Rutgers genetic map^[24]^. For each set of simulated genotypes, the SGS sharing is determined and optimal null segments across the genome established in parallel process to that performed in the real data. The nominal empirical *P*-value for an observed segment is the proportion of null optimal segments at the same position that are the same or longer than that observed. At one million simulations, a distribution is fit, based on the set of genome-wide empirical *P*-values (under the assumption that the majority of segments across the genome represent the null). This distribution is used to establish the pedigree-specific genome-wide threshold, corresponding to a false-positive rate of μ = 0.05 per genome, based on the Theory of Large Deviations^[25]^. Simulations then continue, as necessary, until all *P*-values are estimated to resolution.

### Establishing germline sharing

The DNA studied was derived from whole blood lymphocytes and therefore may be contaminated with malignant CLL cells. To delineate possible contamination, we obtained second blood draws for two of the CLL cases and used flow cytometry to cell-sort CD19+/CD5+ cells (malignant CLL cells) and non-malignant cells (reflective of germline). Genotypes from these sorted cells were used to confirm that alleles shared in SGS regions were germline in origin.

### Haplotype estimation

At a locus, SGS analysis identifies the region with the best statistical evidence (lowest *P*-value) and defines the subset of cases that share it (the sharing group). By definition, all cases in the sharing group can share a haplotype across the best region. Subsets of the sharing group may, however, share longer regions (with less significant *P*-values). We followed the pattern of *P*-values as they iteratively diminished to identify the longer segments shared by fewer cases in the sharing group. Cases who are removed from subsequent longer regions indicate loss of the ancestral haplotype, i.e., a recombinant event. In this way, the haplotypes for each individual of the sharing group can be estimated surrounding the SGS region.

### Human Protein Atlas transcriptome analysis

We used three publicly available datasets from the Human Protein Atlas (HPA) version 20.0 (https://v20.proteinatlas.org/) to examine the expression for genes in an SGS region in the most relevant tissues, cell-lines and cell types from peripheral blood mononuclear cells^[26–29]^. Expression data for 37 tissues, 69 cell lines (no CLL) and 18 blood cell types were available. Normalized expression values for five lymph tissues (B-cells, bone marrow, lymph node, spleen and T-cells), seven cell-lines [Daudi (human Burkitt lymphoma), Karpas-707 (multiple myeloma), REH (pre-B cell leukemia), RPMI-8226 (multiple myeloma), U-266/70 (multiple myeloma, IL-6-dependent), U-266/84 (multiple myeloma), U-698 (lymphoblastic lymphosarcoma)], and two blood cell types (memory B-cells, naïve B-cells) were selected as most relevant.

## RESULTS

All eight sampled CLL individuals in the pedigree passed genotyping quality control. The final pedigree for analysis included the eight CLL cases separated by 28 meioses. A genome-wide significance threshold of α = 3.94 × 10^−7^ was established for the pedigree.

**Figure 2.**
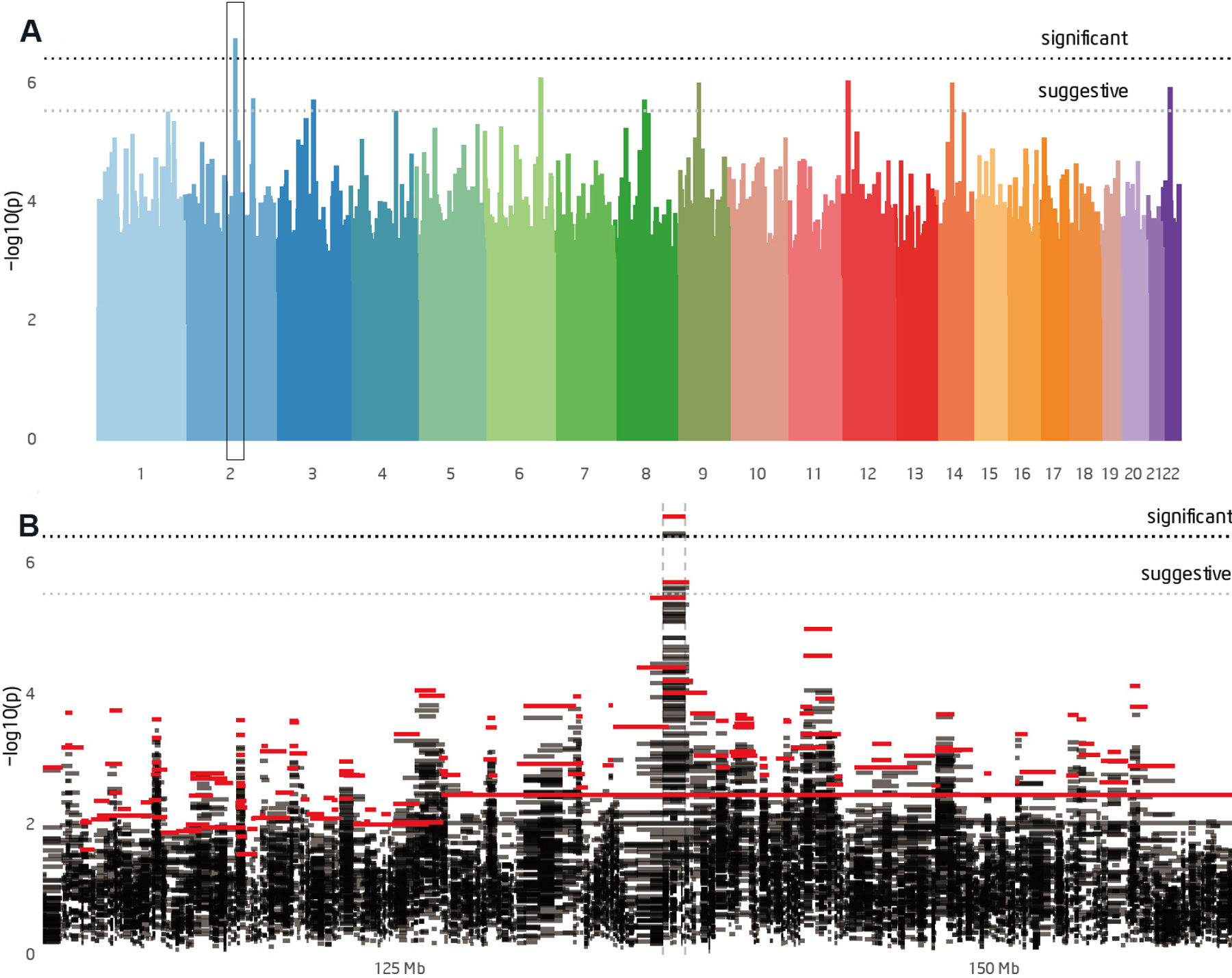
Shared genomic segment (SGS) analysis results. (A) Manhattan plot of the genome-wide SGS optimal segment *P*-values. Significant threshold (μ = 0.05) is 3.98 × 10^-7^. Suggestive threshold (μ = 1.0) is 2.98 × 10^-6^. (B) SGS segment plot focused on the 50 Mb surrounding the significant peak at 2q22.1. The plot shows all the SGS segments and their *P*-values. Segments in the optimal set (segments that are the most significant *P*-value at a position in the genome) are highlighted in red.

**Figure 3.**
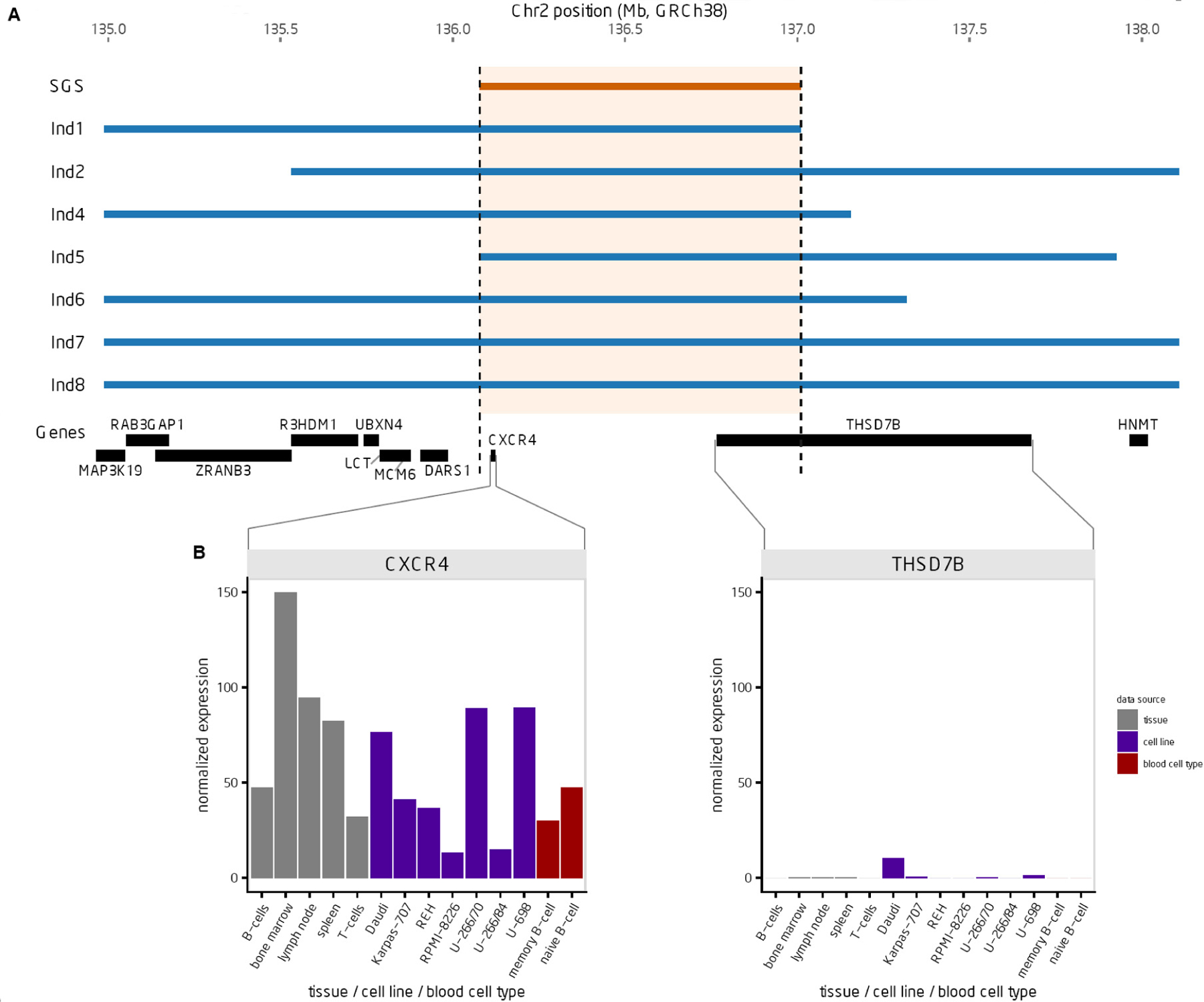
Characterization of the shared genomic segment (SGS) region (A) SGS region, seven estimated haplotypes (inherited from the common founder), and location of genes in the region. (B) Expression of *CXCR4* and *THSD7B* using data from the Human Protein Atlas for 14 relevant tissues/cell lines/blood cell types.

One genome-wide significant SGS region was identified at chromosome 2q22.1 (*P* = 1.9 × 10^−7^, LOD-equivalent 5.6) [Figure 2A and B]. This 2q22.1 locus is inherited through 26 meioses to seven of the eight studied CLL cases [Figure 1B]. Two additional obligate carriers (parents) with hematological malignancies also shared the segregating region: non-Hodgkin lymphoma, NOS and leukemia, NOS [Figure 1B]. The region shared by all seven CLL cases contains 204 consecutive SNPs and is 0.9 Mb in length, from 136.1–137.0 Mb (GRCh38). Alleles in the sorted cells confirmed the shared region was germline. Figure 3A illustrates the SGS region and each of the seven estimated haplotypes in the case-sharers at this locus. The shared region encompasses the entire *CXCR4* gene, part of the gene, *THSD7B* (thrombospondin type 1 domain containing 7B), and two unstudied non-coding genes (*AC112255.1* and *RN7SKP141*). The mRNA expression of *CXCR4* and *THSD7B* in 14 relevant tissues, cells and cell-lines from the HPA is shown below each gene [Figure 3B]^[28]^. Expression of *CXCR4* was evident in all 14 relevant tissues/cell-lines/cells, and highest in bone marrow and lymph node. Expression of *THSD7B* was virtually nonexistent in all tissues/cells [Figure 3B].

## DISCUSSION

Despite the consistent and significant evidence for familial clustering in CLL, the rarity of the malignancy and its etiologic complexity have challenged discovery of segregating risk genes in families. Early linkage studies did not identify any significant loci^[5,6,8,9]^. The largest collaborative study including 206 mostly nuclear families identified one significant locus (*P* = 7.7 × 10^−5^)^[7]^. With many small pedigrees, a 1-LOD support interval surrounding the peak is standard for localization, identifying the region that has odds within an order of magnitude of the peak. This identifies chromosome 2.2q21.2–2q22.1 as the localized region. This ~6.25 Mb region harbors 18 protein-coding genes (GRCh38) including, as noted by the authors, *CXCR4* as the likely candidate. Our study of a large high-risk CLL pedigree represents the largest single pedigree studied to-date. We identified one genome-wide significant finding (*P* = 1.9 × 10^−7^), a 0.9 Mb region that lies within the previously suggested ~6.25 Mb region. Our result replicates and substantially narrows the locus, which now implicates only two genes: *CXCR4* and *THSD7B*.

Overlay of expression in relevant tissues point to *CXCR4* as the compelling candidate [Figure 3B]. Further, there is a rapidly growing literature on *CXCR4* in CLL, while in contrast no published articles exist for *THSD7B* and CLL. *CXCR4* has been shown to be overexpressed in malignant CLL cells^[30]^, and has been associated with disease progression^[31,32]^, and Rai stage^[33]^ as well as worse prognosis^[34]^ and survival in familial CLL^[35]^. The 5’ UTR of *CXCR4* has been shown to be recurrently mutated in CLL^[36]^ and has been found as a proto-onco fusion gene with *MAML2*^[37]^. It been additionally been shown to be a key molecule in CLL cell trafficking into and out of the bone marrow^[38]^, referred to as “*CXCR4*-mediated migration”, and influential in dependencies with the microenvironment^[39]^. Given its vital function in CLL proliferation, targeting *CXCR4* in CLL has shown efficacy in treating the disease as well as modifying drug-response, particularly with the drug ibrutinib^[40–45]^. Additionally, responses to ibrutinib were influenced by somatic *MYD88* and *CXCR4* mutations in patients with Waldenström’s macroglobulinemia^[46]^.

In addition to CLL, *CXCR4* is overexpressed in over 23 cancers, including lung, prostate, melanoma, and uterine cancers (reviewed in^[47,48]^). Four of the CLL SGS sharers were also diagnosed with solid cancers: two melanoma, two prostate, urinary systems, and head and neck cancer [Figure 1B]. Three obligate carriers were also diagnosed with solid cancers: prostate, gastrointestinal, gynaecological, and lung cancer [Figure 1B]. The only published article with *THSD7B* and any cancer is a GWAS study that identified a common variant (rs13405020, *P* < 7 × 10^−6^) outside of the SGS region in *THSD7B* in Korean patients with non-small cell lung cancer^[49]^.

A small case-control gene-panel sequencing study of sporadic CLL included *CXCR4*, and identified a common variant in *CXCR4* (rs2228014, MAF = 0.04) that was increased in CLL cases (uncorrected *P* = 0.0015)^[50]^. The association of this variant with CLL was not replicated in a second, larger case-control study (*P* = 0.84)^[51]^. However, one rare truncating variant and one missense variant were observed in *CXCR4* in two CLL cases with positive family history which was absent from controls (not statistically significant)^[51]^. Truncating germline mutations in the C-terminus of *CXCR4* have been shown to act as gain-of-function mutations and cause WHIM syndrome (warts, hypogammaglobulinemia, infections, and myelokathexis), and Waldenström’s macroglobulinemia^[52]^.

Our analysis was limited to the autosome, a restriction of the current SGS algorithm. None of the six previously proposed CLL risk genes from direct-to-sequencing or CNV analyses in family-based designs are located on the sex chromosomes. These are *ITGB2*^[10]^, *POT1*, *TERF2IP*, *ACD*^[11]^, *DLEU7* and *IRF4*^[13]^. All remain to be replicated. Attempts to replicate recurrence of *ITGB2* rs2230531 in families^[12]^ or association in sporadic case-control designs have not been successful^[53]^. We did not find significant or suggestive evidence of segregation of any of these loci in our pedigree, although we are limited by investigating only one family.

In summary, we have studied a single six-generation high-risk CLL pedigree and identified a genome-wide significant region at 2q22.1 shared by seven CLL cases and two obligate carriers with hematological malignancies. The 0.9 Mb region replicates and narrows a previously proposed linkage locus for CLL. In a complex field which has lacked in replication of family-based findings thus far, this result is extremely encouraging. Within the shared region, *CXCR4* is a compelling candidate. The seven haplotype carriers in the pedigree provide a valuable resource for pursuing the functionally relevant variant/s (coding or regulatory) that reside on the shared haplotype. Future work will elucidate if, in fact, *CXCR4* plays a role in inherited risk, as implicated here.
